# Adherens Junction Integrity Is a Critical Determinant of Sodium Iodide Symporter Residency at the Plasma Membrane of Thyroid Cells

**DOI:** 10.3390/cancers14215362

**Published:** 2022-10-31

**Authors:** Márcia Faria, José Vareda, Micaella Miranda, Maria João Bugalho, Ana Luísa Silva, Paulo Matos

**Affiliations:** 1 Department of Endocrinology, Diabetes and Metabolism, Hospital Santa Maria-Centro Hospitalar Universitário de Lisboa Norte, 1649-028 Lisboa, Portugal; 2 BioISI-Biosystems and Integrative Sciences Institute, Faculdade de Ciências, Universidade de Lisboa, 1749-016 Lisboa, Portugal; 3 Departamento of Human Genetics, Instituto Nacional de Saúde Doutor Ricardo Jorge, 1649-016 Lisboa, Portugal; 4 ISAMB-Instituto de Saúde Ambiental, 1649-028 Lisboa, Portugal; 5 Faculdade de Medicina, Universidade de Lisboa, 1649-028 Lisboa, Portugal

**Keywords:** thyroid cancer, RAI-refractory, NIS, adherens junctions, plasma membrane localization

## Abstract

**Simple Summary:**

Most cases of differentiated thyroid carcinoma (DTC) are associated with a good prognosis. However, a significant number progress to advanced disease exhibiting aggressive clinical characteristics. These cases have a poorer prognosis because they become resistant to radioactive iodine (RAI) treatment. One of the causes for this resistance is the reduction of the channel responsible for iodide uptake (NIS—the sodium iodide symporter) at the plasma membrane (PM) of metastatic thyroid cancer cells. Here we describe that cell–cell adhesion is a key determinant for NIS residency at the PM, suggesting that loss of cell–cell adhesion during metastization contributes to RAI treatment resistance in advanced TC. Our findings indicate that successful resensitization therapies might require the use of agents that improve epithelial cell–cell adhesion in refractory TC cells.

**Abstract:**

While most cases of differentiated thyroid carcinoma (DTC) are associated with a good prognosis, a significant number progress to advanced disease exhibiting aggressive clinical characteristics and often becoming refractory to radioactive iodine (RAI) treatment, the current gold-standard therapeutic option for metastatic disease. RAI-refractoriness is caused by defective functional expression of the sodium-iodide symporter (NIS), which is responsible for the active transport of iodide across the plasma membrane (PM) into thyroid follicles. NIS deficiency in these tumors often reflects a transcriptional impairment, but also its defective targeting and retention at the cells’ PM. Using proteomics, we previously characterized an intracellular signaling pathway derived from SRC kinase that acts through the small GTPase RAC1 to recruit and bind the actin-anchoring adaptor EZRIN to NIS, regulating its retention at the PM of both non-transformed and cancer thyroid cells. Here, we describe how by reanalyzing the proteomics data, we identified cell–cell adhesion as the molecular event upstream the pathway involved in the anchoring and retention at the PM. We show that by interacting with NIS at the PM, adherens junction (AJ)-associated P120-catenin recruits and is phosphorylated by SRC, allowing it to recruit RAC1 to the complex. This enables SRC-phosphorylated VAV2 exchange factor to activate RAC1 GTPase, inducing NIS retention at the PM, thus increasing its abundance and function at the surface of thyroid cells. Our findings indicate that the loss of epithelial cell–cell adhesion may contribute to RAI refractoriness, indicating that in addition to stimulating NIS expression, successful resensitization therapies might require the employment of agents that improve cell–cell adhesion and NIS PM retention in refractory TC cells.

## 1. Introduction

Differentiated thyroid carcinoma (DTC) is the most frequent endocrine malignancy, accounting for 85–95% of all thyroid cancers (TCs). Despite its increasing incidence, most DTC are associated with a good prognosis and can be effectively managed either by surgery alone or through a combination of surgery and radioactive iodine (RAI) treatment [1,2]. RAI is the first-line therapy for DTC metastatic disease and is still the most effective treatment available [3]. However, a significant number of DTCs progress to advanced disease exhibiting aggressive clinical characteristics and several of them become resistant to current therapeutic options [1,2]. In fact, a significant proportion of advanced metastatic DTC fail to uptake sufficient iodide and to respond to RAI (refractory-DTC). On the other hand, chemotherapy is for the vast majority of DTC ineffective. For these patients, Tyrosine Kinase Inhibitors (TKI) may be considered. This approach is associated with variable responses, significant costs, adverse effects and often leads to drug resistance [3]. It is therefore clinically relevant to develop strategies aiming to increase iodide uptake in refractory-DTC, so as so to enable the treatment with RAI.

The active transport of iodide across the plasma membrane (PM) into thyroid follicles is mediated by NIS, an intrinsic basolateral PM protein of thyroid follicular cells [4]. While NIS levels and iodine uptake are reduced in TC when compared to normal tissue [5], the retention of the functional expression of NIS in most DTCs enables RAI therapy [6]. Defective functional NIS expression is thus the main reason for impaired iodide uptake leading to RAI-refractory TC [6,7]. This reflects a combination of both NIS transcriptional impairment and defective NIS-PM targeting and retention.

Although several preclinical and clinical studies have illustrated the clinical relevance of improving NIS overall expression to enhance the efficacy of RAI therapy, no complete responses have been demonstrated for any agent tested [6]. It is also worth noting that in many RAI-refractory thyroid cancers, NIS is still expressed or even sometimes overexpressed, but it is mainly localized in the cytoplasm, where it is unable to transport iodide [8,9]. It is thus becoming clear that to achieve an efficient response to RAI therapy in refractory-TC, approaches directed at upregulating NIS expression at mRNA/protein level will be ineffective if not combined with additional strategies promoting NIS posttranslational abundance at the PM [7]. Finding new targets to render a functional NIS protein at the membrane is emerging as a requirement for overcoming iodine refractoriness in TC. Since little is known about the mechanisms regulating NIS PM residency, in a previous work, we employed a new experimental strategy to purify and characterize proteins which are selectively associated with NIS at the PM, potentially modulating its abundance and stability on the surface of TC cells [10].

We identified a set of NIS interactors involved in the regulation of the actin cytoskeleton, from which it was possible to characterize an intracellular signaling pathway derived from SRC kinase, which, acting through RAC1, leads to the recruitment and binding of the actin-anchoring EZRIN to NIS, promoting its retention at the PM of thyroid cells [10].

In the present study, our aim is to clarify the signaling cues behind SRC activation that ultimately leads to RAC1 activation and RAC1-mediated retention of NIS at the PM. Reanalysis of the previously collected datasets of NIS interactors at the PM revealed “adherens junctions” (AJs) as another overrepresented pathway. Following this clue, we identified AJ-associated P120 catenin as a pivotal protein to mediate the recruitment of NIS, SRC kinase and RAC1 to a PM-localized complex where the activation of RAC1 by SRC-phosphorylated VAV2 enables the retention of NIS at the cells’ surface. These findings highlight the compromise of cell–cell adhesion through epithelial dedifferentiation in advanced TC as a critical determinant for RAI refractoriness, suggesting that successful resensitization therapies might require the combination of agents that improve epithelial differentiation with compounds stimulating NIS expression.

## 2. Materials and Methods

### 2.1. Cell Lines and Culture

HA-NIS-TPC-1- cells were established from the human PTC-derived cell line TPC-1, modified to stably express the NIS construct containing an extracellular triple HA tag and co-express the halide-sensitive yellow fluorescent protein YFP-F46L/H148Q/I152L (HS-YFP), as previously described [10]. Cells were maintained in 10% *v/v* FBS-supplemented RPMI (Gibco, Grand Island, NY, USA).

The also previously described Y-PCCL3 cells, constitutively expressing the halide sensor HS-YFP [11], were cultured in F12M medium (F-12 Coon’s modified medium (Merck, Darmstadt, Germany), supplemented with thyroid stimulating hormone (0.1 mU/mL, TSH), Apo-Transferrin (5 μg/mL, Apo-T) and insulin (10 μg/mL) (all from Sigma-Aldrich, St. Louis, MO, USA) and Fetal Bovine Serum (5%, FBS, Gibco, Grand Island, NY, USA)). When required, cells were maintained in stimulation medium (F12M supplemented with additional TSH (1 mU/mL)) or starvation medium (F12M without TSH).

All cells were regularly checked for absence of mycoplasm infection and maintained up to 20 passages at 37 °C in a 5% humidified CO_2_ environment.

Plasmid pCDNA3-MYC-RAC1-V12 [11] transfection into HA-NIS-TPC1 cells was performed as previously described [10].

RNA interference experiments were performed in cells seeded in 35 mm dishes using Lipofectamine 2000 and 100 pmol of specific pre-designed siRNAs purchased from Santa Cruz Biotechnology (Santa Cruz, CA, USA): P120 (sc-36139) and Vav2 (sc-41738). The siRNA oligos against firefly luciferase gene (LUC; 5′-CGU ACG CGGAAU ACU UCG ATT) (Eurofins Genomics Ebersberg, Germany)) were used as a control siRNA.

Treatment of cells with the SRC Inhibitor PP2 (2 µM, Sigma-Aldrich, St. Louis, MO, USA) was performed for 1 h in the appropriate medium. Cells treated with the same volume of solvent (vehicle) were used as control.

### 2.2. Cell Surface Protein Biotinylation, CRIB Pull-Down and Co-Immunoprecipitation Assays

Cell surface protein biotinylation assay in TPC1 and PCCL3 cells was performed as described [10,12]. Briefly, cells were incubated with ice-cold PBS++ (PBS pH 8.0 containing 0.1 mM CaCl_2_ and 1 mM MgCl_2_) to arrest the endocytic traffic and cell surface proteins were labeled with sulfosuccinimidyl 3-[[2-(Biotinamido)ethyl] di-thio] pro-pionate sodium salt (0.5 mg/mL, sc-212981, Santa Cruz Biotechnology, Santa Cruz, CA, USA). Then, cell surface proteins were captured with streptavidin-conjugated agarose beads (Sigma-Aldrich, St. Louis, MO, USA). CRIB-pull-down assays for RAC1 activation status were performed as described previously [10].

Co-immunoprecipitation of plasma membrane-specific NIS interactors in HA-NIS-TPC1 cells was performed as previously described [10]. Briefly, as for cell surface protein biotinylation assays, to ensure arrest of endocytic traffic, cells were incubated in ice-cold PBS++ and then cell surface-associated HA-NIS proteins were selectively captured with 4 µg/mL of rabbit anti-HA (H6908; Sigma-Aldrich, St. Louis, MO, USA) coupled to Dynabeads G-protein beads (Invitrogen). Rabbit anti-goat IgG was used as a control condition (5160-2104; BioRad, Hercules, CA, USA).

In all different assays, cell lysates representing input protein levels were also collected. Input and pulled-down protein fractions were solubilized in 2x modified Laemmli buffer (62.5 mM Tris/HCl pH 6.8, 3% (*v*/*v*) SDS, 10% (*v*/*v*) glycerol, 0.02% (*v*/*v*) bromophenol blue, 296.4 mM dithiothreitol (DTT)), and then analyzed by Western blot.

### 2.3. Western Blot

Western blots were performed using standard protocols in 10% SDS-PAGE and transferred to PVDF membranes (Bio-Rad, Hercules, CA, USA). Primary antibody and dilutions used were the following: mouse monoclonal anti-HA (11 583 816 001, Roche, Mannheim, Germany); rabbit polyclonal anti-NIS (24324-1-AP, Protein-tech, Rosemont, IL, USA); mouse monoclonal anti-PCNA (NA03, Merck, Darmstadt, Germany); rabbit anti-GLUT1 (ab652; Abcam, Cambridge, UK); goat anti-GLUT1 (sc-1603; Santa Cruz Biotechnology, Santa Cruz, CA, USA); mouse monoclonal anti-RAC1 (05-389, Millipore, Burlington, MA, USA); mouse monoclonal anti-SRC antibody (60315-1-lg, Proteintech, Rosemont, IL, USA); rabbit anti-phospho Y416-SRC antibody D49G4 (#6943, Cell Signaling, Danvers, MA, USA); mouse monoclonal anti-VAV2 antibody (sc-271442, Santa Cruz Biotechnology, Santa Cruz, CA, USA); rabbit polyclonal anti-phospho Y172-VAV2 antibody (ab86695, Abcam, Cambridge, UK); mouse monoclonal anti-P120 catenin antibody 15D2 (#33-9600, Thermofisher, Waltham, MA, USA); rabbit polyclonal anti-phospho Y228-P120 catenin (329050; US Biological, Salem, MA, USA). HRP-labeled anti-rabbit or anti-mouse IgG secondary antibodies (Bio-Rad, Hercules, CA, USA) were used for chemiluminescence imaging.

### 2.4. HS-YFP–Based Iodide Influx Assay

Iodide (I-) influx was performed as previously described in both HA-NIS-TPC1 and PCCL3 cells stably expressing the halide-sensitive yellow fluorescent protein (HS-YFP- F46L/H148Q/I152L) [10]. Briefly, cells were plated on 8-well chamber slides (Ibidi, Gräfelfing, Germany) and iodide influx was assayed after addition of isomolar PBS-NaI solution (final NaI concentration of 50 mM for TPC1 and 1 mM for PCCL3 cells) by recording fluorescence continuously for 500 s (acquiring an image every 10 s) in a Leica fluorescence microscope. Fluorescence quantification was performed in stacked images using Image J (NIH, Bethesda, Maryland, USA) as previously described [10,11,13] and iodide influx rates were extrapolated by fitting the obtained curves of fluorescence decay to exponential functions, also as described [10,11,13]. Specificity of NIS-mediated iodide uptake was controlled by performing the assay in the presence of ClO_4_^−^ (PCCL3, 1 mM; TPC1, 50 mM), a potent competitive inhibitor of NIS.

### 2.5. Immunoflurescence and Confocal Microscopy

HA-NIS-TPC1 cells were cultured in coverslips and treated as indicated and fixed for 30 min in PBS containing 4% formaldehyde (Sigma-Aldrich, St. Louis, MO, USA). Cells were then permeabilized for 10 min with 0.2% Triton X-100 (Sigma-Aldrich, St. Louis, MO, USA) in PBS, washed trice with PBS, and incubated for 1 h in PBS containing 0.1% Tween 20 (Sigma-Aldrich, St. Louis, MO, USA) together with 2 µg/mL of rabbit anti-HA (H6908; Sigma-Aldrich, St. Louis, MO, USA) and 1 µg/mL of mouse anti-E-cadherin (610181; BD Biosciences, Franklin Lakes, NJ, USA). After extensive PBS washing, cells were further stained with phalloidin-FITC (Sigma-Aldrich, St. Louis, MO, USA), Alexa Fluor 405- and Alexa Fluor 546-conjugated goat anti-mouse and goat anti-rabbit, respectively (both from Thermofisher, Waltham, MA, USA). Coverslips were mounted with Everbrite mounting medium (Biotium, Fremont, CA, USA) and fluorescence images were acquired with a Leica TCS SPE confocal microscope.

### 2.6. Reanalysis of MS Data and Statistical Analysis

The selection of high-confidence NIS-interactor candidates among the proteins identified in our previous study followed the analytic pipeline described therein [10]. Functional enrichment analysis among the selected high-confidence candidate datasets was performed using STRING software (https://string-db.org/, accessed on 5 October 2021), covering Gene Ontology (GO), Kyoto Encyclopedia of Genes and Genomes (KEGG) databases. STRING was also used to construct protein interaction networks and generate protein interaction maps.

GraphPad Prism 5 software was used for general statistical analysis. The shown results are means ± standard error of the mean (SEM) from at least three independent experiments. For multiple comparisons, one-way ANOVA tests were applied followed by post-hoc Tukey HSD or Dunnett’s tests. To compare two sets of data, we employed unpaired Student’s t-tests. The significance level was set at 0.05 in all analyses ((*) *p* < 0.05; (**) *p* < 0.01; (***) *p* < 0.001).

## 3. Results

### 3.1. Reanalysis of Proteomic Data Highlighted the Adherens Junction Pathway as the Second Best Represented in the Dataset of NIS PM Interactors

As mentioned above, we previously used a proteomics approach to characterize what proteins associated with NIS specifically at the PM of thyroid cells [10]. We found these NIS-PM-exclusive interactors to be highly enriched in proteins associated with the organization of the actin cytoskeleton and, consistently, KEGG analysis highlighted several proteins belonging to the “Regulation of actin cytoskeleton” pathway within the dataset [10]. Biochemical and functional analysis allowed us to establish the SRC/RAC1/PAK1/PIP5K/EZRIN pathway as a key regulator of NIS actin-cytoskeleton anchoring and retention at the PM [10]. However, at that point, it was still unclear which molecules mediated RAC1 activation downstream of SRC and what signals were determining SRC activation and the recruitment of NIS and RAC1 to the same PM location to enable the latter to promote the anchoring and retention of the former. This prompted us to reanalyze the data using not only the 109 NIS-PM-exclusive interactors, but also including the 33 proteins that appeared in both the NIS-PM and NIS-whole cell lysates datasets. STRING platform-based analysis revealed that whereas “Regulation of actin cytoskeleton” was still the top overrepresented KEGG pathway within the dataset (Table 1), “Adherens junction” was a close second, with an identical confidence score (FDR = 1.84 × 10^−6^, in both cases). In addition, while several hit proteins, including SRC and RAC1, were annotated to the two pathway categories, others were pathway specific, including P120-catenin (CTNND1), which appeared associated only with adherens junctions (AJ) (see Supplementary Figure S1).

### 3.2. Adherens Junction Integrity Influences NIS PM Abundance

**Figure 1:**
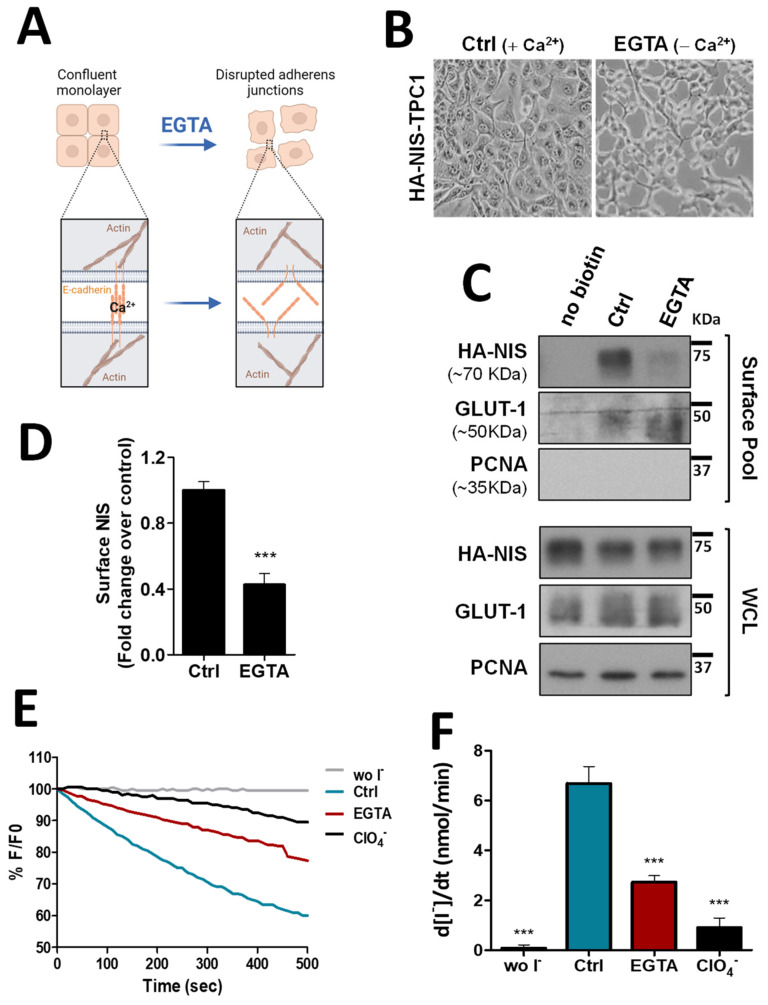
Impact of adherens junction integrity on HA-NIS abundance and function at the PM. (**A**) Representative diagram of AJs disruption by EGTA, a chelator with specificity for calcium ions, that destabilizes AJs by removing calcium from interacting cadherins in adjacent cells. AJ dissociation leads to the disaggregation of the catenin-mediated complex that anchors the AJ to actin. The figure was created with BioRender.com, accessed on 26 July 2022. (**B**) Representative images of AJs disruption of HA-NIS-TPC1 cells treated for 30 min with either CaCl_2_ (1 mM)-containing PBS (Ctrl) or calcium-free PBS containing 4 mM of EGTA. (**C**) HA-NIS PM abundance was assessed in cells treated as in (**B**) by surface protein biotinylation. Surface fraction or the correspondent whole-cell lysates (WCL) were analyzed by WB using an anti-HA antibody to detect the HA-NIS protein. A replica where cells treated as in ‘Ctrl’ were not incubated with biotin (no Biotin) controlled the specificity of biotinylated protein pull-down. GLUT-1 served as loading control (for densitometric normalization) among cell-surface protein extracts and WCL, whereas PCNA monitored for both WCL loading and eventual intracellular protein contamination in cell-surface extracts. (**D**) Surface HA-NIS abundance was quantified by densitometry analysis of WB bands, using ImageJ software. Plotted values are means ± SEM of three independent assays. Comparisons to each corresponding control condition were made using a two-tailed Student’s *t*-test (*** *p* ≤ 0.001). (**E**) HA-NIS-TPC1 cells stably expressing the YFP halide sensor (HS-YFP) were used to analyze the HA-NIS protein function. The decay of the YFP fluorescence in cells treated as in (**B**) was monitored continuously for 500 s, acquiring an image every 10 s, after exposing the cells to 50 mM NaI. The presence of a competitive inhibitor of iodide uptake by NIS (ClO_4_^−^-; 50 mM for 10 min) slows or prevents the decay of YFP fluorescence over time, confirming assay specificity. (**F**) Iodide influx rates were derived from the decay curves. Data are means ± SEM of three independent assays. Significances were assessed by one-way ANOVA (F = 49.63; *p* < 0.001) followed by Dunnett’s tests, compared to control conditions (Ctrl) (*** *p* < 0.001). The uncropped bolts are shown in Supplementary Materials File S1.

To validate the biological significance of this new analysis, we took confluent TPC1 thyroid cells, stably expressing an extracellularly tagged HA-NIS protein (HA-NIS-TPC1), and employed a calcium depletion assay to destabilize cadherin-based AJ to determine whether the loss of AJ integrity would impact NIS abundance at the cells’ PM. These cells were the model originally used to gather the proteomics data [10] and thus the adequate starting point to validate their analysis. For this, the cells’ culture medium was replaced for 30 min with calcium-free PBS containing 4 mM of ethylene glycol tetraacetic acid (EGTA), a chelator with specificity for calcium ions, that destabilizes AJs by removing calcium from interacting cadherins in adjacent cells, leading to their dissociation and the disaggregation of the catenin-mediated complex that anchors the AJ to actin [14] (see diagram in Figure 1A and representative images in Figure 1B). Control and EGTA-treated cells were then placed on ice and incubated with NHS-SS-biotin, to selectively label proteins at the cell surface. Labeled proteins were captured with streptavidin beads (surface protein fraction—PM) and the abundance of PM-associated NIS compared to whole cell lysates (WCL) was analyzed by Western blot (WB). As shown in Figure 1C (quantified in Figure 1D), NIS abundance at the cells’ PM was decreased by two-fold upon AJ dissociation with EGTA, whereas its total levels remained constant. In addition, we performed immunofluorescence staining of these cells and observed that whereas in the presence of Ca^2+^ there was NIS localizing with actin and E-cadherin at cell–cell contacts, EGTA treatment led to cell scattering, cadherin internalization and removal of NIS from the cell periphery, some of which re-localized to the cytoplasm together with internalized E-cadherin (see Figure S2). These observations were consistent with AJ integrity being required to sustain NIS permanence at the PM of thyroid cells. To further validate these findings, we repeated these experiments in TPC1-HA-NIS cells co-expressing the halide-sensitive mutant of yellow fluorescent protein (YFP) F46L/H148Q/I152L (HS-YFP), which is quenched by iodide, and that we had previously used successfully to study NIS-mediated iodide transport in these and other thyroid cells [10,12]. As shown in Figure 1E,F, disruption of AJ integrity through EGTA treatment led to a decrease in the rate of NIS-mediated iodide influx (confirmed by its blocking in the presence of NIS inhibitor ClO_4_^−^) comparable to the observed decrease in NIS PM levels (Figure 1C,D).

**Figure 2:**
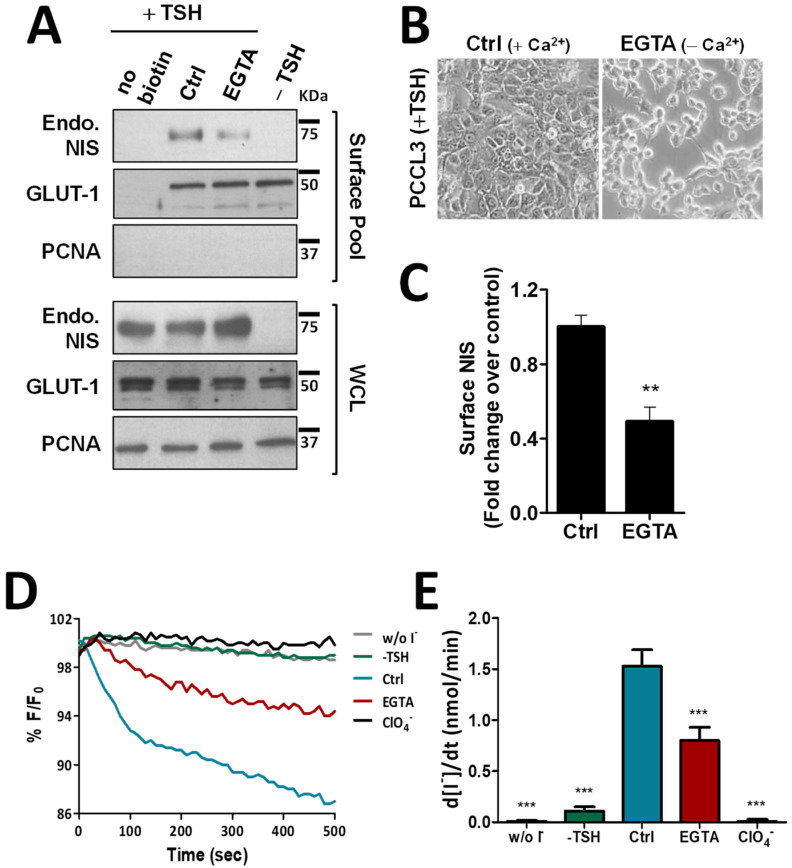
Effect of EGTA-induced AJ disruption on NIS functional expression in non-neoplastic, TSH-responsive thyroid follicular cell line. (**A**) PCCL3 cells subjected to a 24 h starvation period (without TSH) followed by stimulation (or not; −TSH) with TSH (1 mU/mL for 48 h; +TSH), were then additionally treated with either CaCl_2_ (1 mM)-containing PBS (Ctrl) or 4 mM of EGTA for 30 min. Endogenous NIS (Endo. NIS) abundance at the PM was assessed by surface protein biotinylation followed by WB using an anti-NIS antibody. Whole-cell lysates (WCL) were also assessed. A replica where cells treated as in ‘Ctrl’ were not incubated with biotin (no Biotin), controlled the specificity of biotinylated protein pull-down. GLUT-1 served as loading control (for densitometric normalization) among cell-surface protein extracts and WCL, whereas PCNA monitored for both WCL loading and eventual intracellular protein contamination in cell-surface extracts. (**B**) Representative images of AJs disruption of PCCL3 cells treated for 30 min with either CaCl_2_ (1 mM)-containing PBS (Ctrl) or calcium-free PBS containing 4 mM of EGTA. (**C**) Surface NIS abundance was quantified by densitometry analysis of WB bands, using ImageJ software. Plotted values are means ± SEM of three independent assays. Comparisons to each corresponding control condition were made using a two-tailed Student’s *t*-test (** *p* ≤ 0.01). (**D**) Traces of HS-YFP fluorescence decay of HS-YFP-PCCL3 cells treated as in (**A**) after exposure to 1 mM NaI, in the presence or absence of ClO_4_^−^ (1 mM, 10 min). YFP fluorescence was recorded continuously, as described in the legend to Figure 1. (**E**) Iodide influx rates were calculated by fitting the curves to the exponential decay curves. The data are the means ± SEM of five independent assays. The significant variations were assessed by one-way ANOVA (F = 49.97; *p* < 0.001) followed by Dunnett’s post-hoc test, as compared with the control conditions (Ctrl) (*** *p* < 0.001). The uncropped bolts are shown in Supplementary Materials File S1.

To exclude that these observations were not limited to this thyroid cancer cell model with NIS ectopic expression, we repeated the above experiments in the non-transformed, follicular thyroid cell line PCCL3. PCCL3 cells respond to TSH stimulation by notably increasing the expression and surface abundance of endogenous NIS protein (Figure 2A). Notably, similar to that observed in HA-NIS-TPC1 cells, upon disruption of AJs by EGTA treatment of TSH-stimulated PCCL3 cells (Figure 2B), the abundance of endogenous NIS at the PM was also significantly reduced (Figure 2A, quantified in Figure 2C), while its overall levels remained unchanged. Likewise, NIS-mediated iodide influx in HS-YFP-PCCL3 cells (abrogated by the presence of inhibitor ClO_4_^−^) was also proportionally reduced by EGTA-induced AJ disruption (Figure 2D,E). These data support that AJ integrity is indeed a requisite to enabling NIS permanence at the PM of thyroid cells.

### 3.3. AJ Disruption Inhibits P120-Catenin-Mediated Activation of VAV2 by SRC in Thyroid Cells

**Figure 3:**
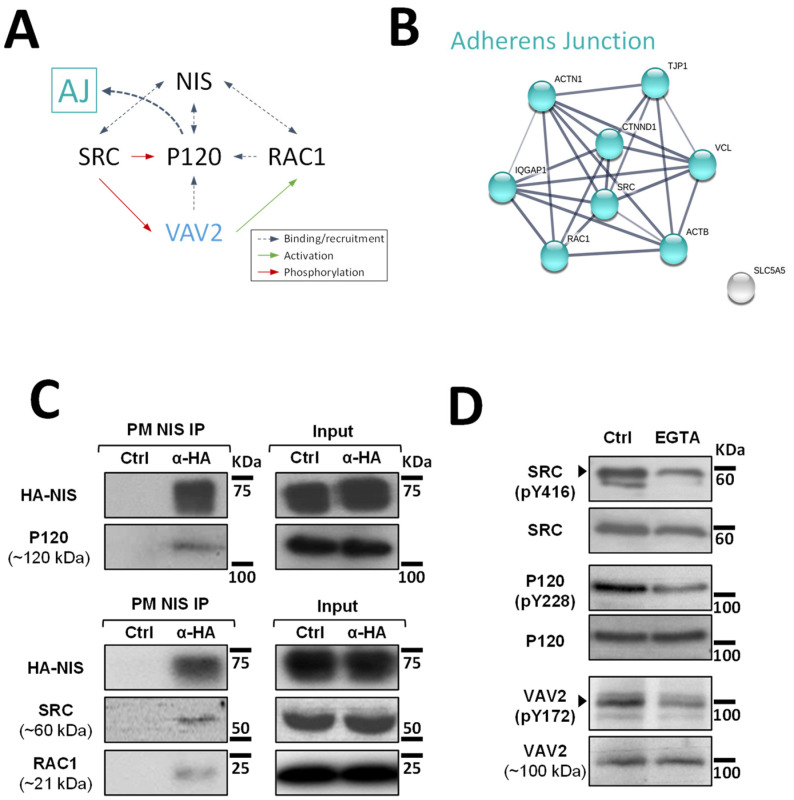
Validation of high-confidence NIS interactions detected by MS analysis. (**A**) Diagram depicting the functional interplay reported in the literature for SRC, RAC1, P120 and VAV2 at adherens junction (AJ) complexes. (**B**) STRING-generated subnetwork with the AJ-associated NIS-PM candidate interactors, detected in our proteomic analysis. The network nodes represent the identified proteins. The grey lines connecting two nodes represent protein associations extrapolated from textmining-, experimental- and database-collected evidence. The thickness of the lines is proportional to the degree of confidence for the predicted association between the nodes. (**C**) NIS-PM immunoprecipitated protein complexes were analyzed by WB, using specific primary antibodies to detect the indicated target candidate proteins. The total (input) and immunoprecipitated (IP) HA-NIS levels were detected using a mouse anti-HA primary antibody to confirm the immunoprecipitation efficiency. (**D**) Impact of EGTA-mediated AJs displacement on SRC, P120 and VAV2 activation status was assessed in HA-NIS-TPC1 cells treated with either vehicle or EGTA (4 mM for 30 min) by monitoring SRC, P120 and VAV2 phosphorylation levels by WB. The uncropped bolts are shown in Supplementary Materials File S1.

Although at this point it was clear that signals originating from AJs promote NIS residency at the PM of thyroid cells, the mechanism that regulates this effect was still undefined. It has been described for other epithelial cells that AJ establishment leads to the recruitment of P120-catenin, which associates with the cadherin juxtamembrane domain to suppress its endocytosis, leading to junction maturation and its anchoring to the cytoskeleton [15]. The latter involves P120-mediated regulation of Rho GTPase activity, namely the activation of RAC1. P120 recruits and becomes phosphorylated by SRC, which allows P120 to directly interact with RAC1 at the PM and to recruit VAV2, one of the RAC-GDP/GTP exchange factors (GEFs) [15,16]. VAV2 is then activated at the AJ by SRC phosphorylation and in turn activates RAC1 (see diagram in Figure 3A), triggering the polymerization of F-actin necessary for AJ anchoring and stabilization [16].

Since P120 (CTNND1) was one of the AJ-associated candidate PM-NIS interactors detected in our proteomic analysis (see Figure 3B and Supplementary Figure S1) and we previously showed that SRC-mediated activation of RAC1 and F-actin polymerization was also necessary for NIS retention at the cell surface [10], we hypothesized that P120 could be the link between AJ signaling and activated RAC1-mediated NIS retention at the cell surface.

To investigate this hypothesis, we started by validating the interaction between P120 and NIS at the cells’ PM. For this, we used the approach we previously described [10] that takes advantage of the extracellular HA tag to selectively mmunoprecipitated NIS-containing complexes from the PM of HA-NIS-TPC1 cells. Using WB, we could readily detect P120 co-precipitating with NIS from the PM (Figure 3C). Moreover, we could also detect SRC and, as described before [10], RAC1 as clear components of NIS-containing complexes at the PM. However, in agreement with the original MS outputs [10], we could not detect VAV2 co-precipitating with PM-NIS. This is not completely unexpected given that the association of GEFs with Rho GTPases at membrane complexes is usually transient, since the GDP to GTP exchange drastically lowers their affinity [17]. Nonetheless, using phosphorylation-specific antibodies (Figure 3D), we could observe that EGTA treatment did lead to a decrease in SRC activation (assessed by autophosphorylation of tyrosine 416 (Y416) required for full catalytic activity [18]). In addition, consistent with VAV2 being recruited by P120 and phosphorylated by SRC at intact AJs in thyroid cells, decreased SRC-mediated phosphorylation of P120 and VAV2 (residues Y228 and Y172, respectively [9]) was also observed upon EGTA treatment.

### 3.4. Adherens Junction Integrity and SRC Activity Are Required to Sustain RAC1 Activation and RAC1-Mediated NIS Residence at the PM

**Figure 4:**
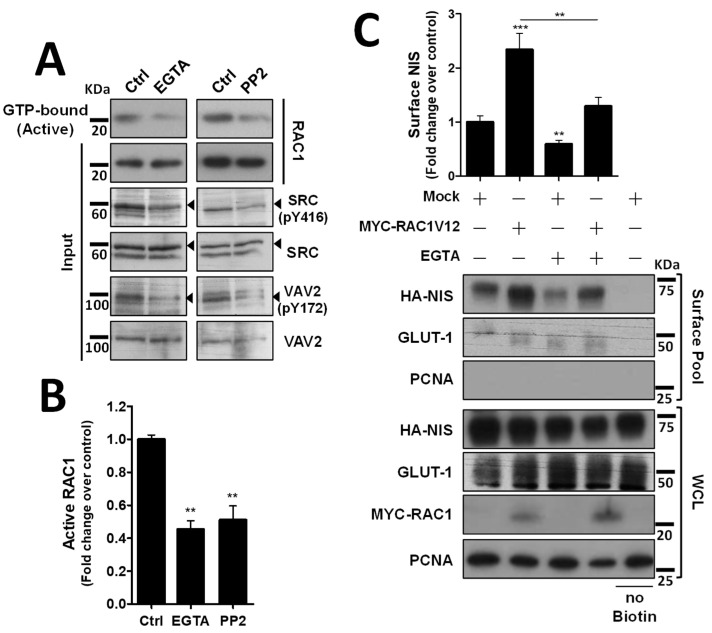
Both AJ integrity and SRC activity impact endogenous RAC1 activation. (**A**) The impact of EGTA-mediated AJ displacement or SRC kinase inhibition on endogenous RAC1 activation status in HA-NIS-TCP1 cells was assessed by monitoring the active, GTP-bound fraction of RAC1 by CRIB domain pull-down assay. Both total (input) and active RAC1 levels were assessed by WB using anti-RAC1 primary antibody. SRC autophosphorylation (using anti-SRC-pY416 antibody) and in SRC activating phosphorylation of VAV2 (using anti-VAV2-pY172 antibody) were also monitored in the same conditions by WB. (**B**) GTP-bound fraction of RAC1 was quantified by densitometry analysis of WB bands, using ImageJ software. Plotted values are means ± SEM of three independent assays. A two-tailed Student’s *t*-test was used to identify significant variations relative to the control (** *p* ≤ 0.01). (**C**) PM-NIS levels were analyzed by surface protein biotinylation in HA-NIS-TPC1 cells treated with EGTA (as in (**A**)) or transfected with either an empty vector (Empty) or GFP-RAC1-V12. Both surface fraction and correspondent whole-cell lysates (WCL) were analyzed by WB, as indicated. A replica where cells treated as in ‘Ctrl’ were not incubated with biotin (no Biotin) controlled the specificity of biotinylated protein pull-down. GLUT-1 served as loading control (for densitometric normalization) among cell-surface protein extracts and WCL, whereas PCNA monitored for both WCL loading and eventual intracellular protein contamination in cell-surface extracts. The WB bands were quantified as in (**B**) and plotted as the means ± SEM of at least four independent assays. Significant differences between treatments were detected using one-way ANOVA analysis (F = 18.09; *p* < 0.001). Significant variations from control conditions (Ctrl) were identified using post hoc Dunnett’s tests identity (** *p* ≤ 0.01; *** *p* ≤ 0.001). The uncropped bolts are shown in Supplementary Materials File S1.

We next investigated how the decrease in VAV2 activation by SRC upon AJ destabilization would affect endogenous RAC1 activation. Using CRIB-pulldown assays, to selectively capture the active, GTP-bound fraction of RAC1 in TPC1 cells, we confirmed that AJ disruption through EGTA treatment caused a clear, over 2-fold decrease in endogenous RAC1 activation in thyroid cells (Figure 4A, quantified in Figure 4B). Moreover, this decrease in RAC1 activation was equivalent to that observed upon direct inhibition of SRC with 2 µM of PP2 inhibitor (Figure 4A,B) and produced a similar reduction in SRC autophosphorylation (SRC-pY416) and in SRC activating phosphorylation of VAV2 (VAV2-pY172), consistent with AJ disruption impairing SRC-induced, VAV2-meditated activation of RAC1 in thyroid cells. In addition, again using cell surface biotinylation, we observed that AJ disruption not only decreased steady-state NIS PM abundance, but completely prevented constitutively active RAC1 (the RAC1-V12 mutant) from upregulating NIS levels at the PM (Figure 4C). This suggests that the complex assembled at AJs is required not only to mediate RAC1 activation, via SRC and VAV2, but also to effectively recruit active RAC1 to the proper location for it to promote NIS retention at the PM.

### 3.5. Preventing RAC1 Recruitment or Activation at AJ Impairs NIS Residency at the PM of Thyroid Cells

**Figure 5:**
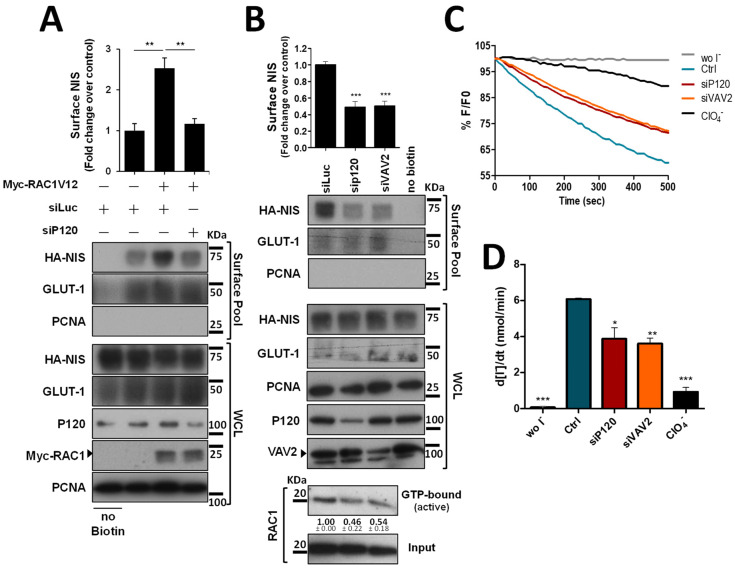
P120 participates in the RAC1-mediated upregulation of NIS PM residency. (**A**) HA-NIS-TPC1 cells were transfected with either an empty vector (Empty) or GFP-RAC1-V12 or with either mock (siLUC control) or a specific siRNA against P120 (siP120), as indicated. (**B**) HA-NIS-TPC1 cells were transfected with either mock (siLUC control), a specific siRNA against P120 (siP120), or a specific siRNA against VAV2 (siVAV2), as indicated. In addition, endogenous RAC1 activation status upon either P120 or VAV2 knockdown was assessed by monitoring the active GTP-bound fraction of RAC1 (using the CRIB domain pull-down assay) and comparing it to total (input) RAC1 levels, using anti-RAC1 primary antibody by WB. NIS-PM levels (**A**,**B**) were assessed by surface protein biotinylation and WB as described in Figure 1, Figure 2 and Figure 4. The plotted values correspond to the means ± SEM of at least three independent assays. One-way ANOVA analysis detected significant differences between the treatments (F = 19.30 and *p* = 0.0024 for (**A**); F = 33.59 and *p* < 0.001 for (**B**)). Significant variations relative to the control conditions or among the different treatments (indicated by horizontal lines) were identified by Tukey’s posthoc tests (** *p* ≤ 0.01; *** *p* ≤ 0.001). (**C**) Representative traces of the iodide-induced YFP fluorescence decay of HA-NIS/HS-YFP-TPC1 cells treated as in (**B**) and analyzed as described in Figure 1. (**D**) Iodide influx rates were derived from the decay curves. Data are the means ± SEM of at least three independent assays. Significant variations were detected by one-way ANOVA (F = 27.48; *p* < 0.001) followed by Dunnett’s posthoc test, compared to control conditions (Ctrl) (* *p* ≤ 0.05; ** *p* ≤ 0.01; *** *p* < 0.001). The uncropped bolts are shown in Supplementary Materials File S1.

Since P120 interacts with both NIS and RAC1 at the PM and was described as the scaffold responsible for the binding and recruitment of RAC1 to AJs [15], we tested the effect of P120 depletion on active RAC1V12-mediated NIS retention at the PM. Similarly to the observed above for EGTA treatment, depletion of P120 completely prevented active RAC1V12 from increasing NIS PM abundance (Figure 5A), indicating that P120 mediates not only RAC1 activation, but also the functional interaction between NIS and RAC1, which enables RAC1 signaling to anchor and retain NIS at the PM (as we have previously shown [10]). Indeed, P120 depletion in thyroid cells was sufficient to downregulate endogenous RAC1 activation and NIS PM abundance to levels comparable to those observed upon interfering with VAV2 (Figure 5B). Moreover, either P120 or VAV2 depletion produced a significant and comparable decrease in NIS-mediated iodide uptake in these cells (Figure 5C, quantified in Figure 5D), consistent with the observed decrease in NIS PM levels.

## 4. Discussion

We previously described that activation of the small GTPase RAC1 downstream of the SRC kinase is a key step towards enabling ezrin-mediated actin-cytoskeleton anchoring and retention of NIS at the PM [10]. However, the signaling cues upstream of SRC leading to the activation of this pathway in thyroid cells remained unclear. Here we reanalyzed the MS data collected in the previous study and identified cell-cell adhesion as a potential candidate stimulus to promote SRC-induced retention of NIS at the PM via localized activation of RAC1 signaling. We were able to show that disruption of AJ in both non-transformed and cancer thyroid cells causes a significant downregulation of NIS PM abundance without changing its overall levels, indicating an impairment of its retention at the cell’s surface. Consistently, this AJ disruption decreases both SRC activity and RAC1 activation in these cells, which we previously demonstrated were sufficient to impair NIS anchoring and retention at the PM [10]. We then showed that this effect was mediated by P120-catenin, which not only co-precipitated with NIS, SRC and RAC1 from the plasma membrane, but also recruited the RAC1 GDP/GTP exchange factor VAV2 that, once phosphorylated and activated by SRC, promoted the activation of RAC1 at the PM enabling NIS retention at the surface. We further showed that recruitment of RAC1 to NIS via P120 was necessary not only to activate the GTPase via SRC/VAV2, but also to enable it to signal for NIS PM retention, since in the absence of P120, constitutively active RAC1-V12 was unable to improve NIS abundance at the cell surface.

**Figure 6:**
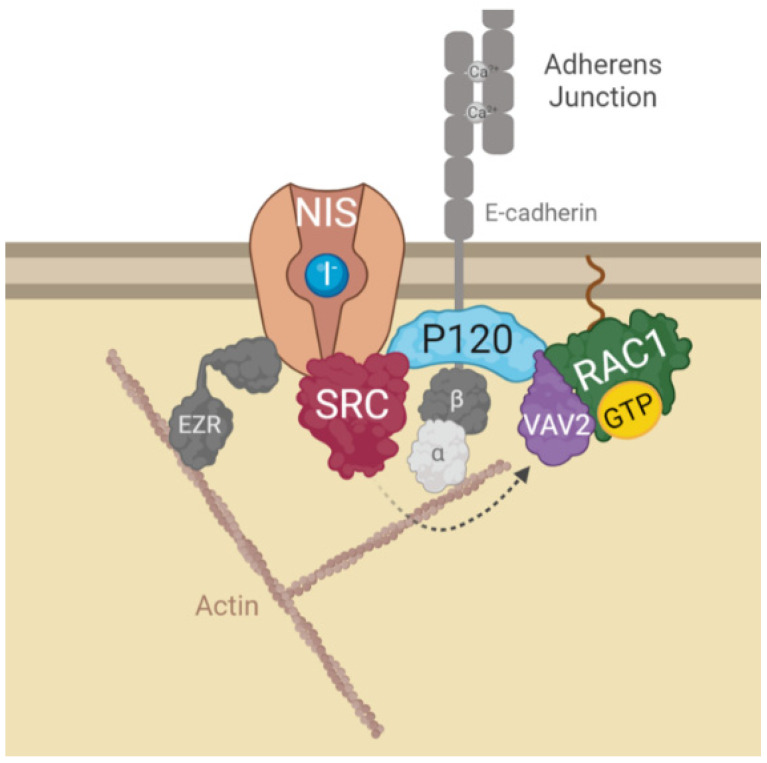
Diagram depicting the model for AJs-derived regulation of NIS functional residency at the PM. In brief, NIS localization and function at the PM depends on its recruitment to AJ complexes at the PM. Interaction of NIS with AJ-localized P120 catenin, allows the assembly of a complex that brings SRC kinase, VAV2 and RAC1 to the correct localization to facilitate the symporter’s retention at the PM. SRC-mediated phosphorylation of the RAC1 GDP/GTP exchange factor VAV2 promotes activation of the small GTPase RAC1 in the vicinity of NIS. RAC1 signaling induces de novo F-actin polymerization and ezrin activation [10], which facilitates the symporter’s functional stabilization at the PM anchoring it to the actin cytoskeleton (see text for further details). The figure was created with BioRender.com, accessed on 26 July 2022.

All these findings led us to propose the model depicted in Figure 6, where the interaction of NIS with AJ-localized P120 catenin, allows the assembly of a complex that brings SRC kinase, VAV2 and RAC1 to the correct localization to facilitate the symporter’s functional stabilization at the PM via anchoring to the actin cytoskeleton, through the localized active RAC1-induced de novo F-actin polymerization and ezrin activation. It should be noted that interference with this mechanism, either through chemical inhibition or depletion of its individual components, significantly reduced, but never completely abolished NIS presence at the cell surface. This suggests that there might be additional mechanisms involved with NIS PM retention that act additively with AJ-mediated signaling to determine the overall functional residency of NIS at the basolateral membrane of thyroid cell in situ. However, our finding that cell–cell adhesions strongly participate in NIS retention at the cell surface contributes to clarify puzzling observations such as why certain RAI-refractory metastatic TCs show nearly absent NIS function despite NIS protein being clearly detectable intracellularly [19]. As in most epithelial tissues, AJs between thyroid cells are mainly mediated by E-cadherin [20]. Several studies have shown that E-cadherin is expressed in normal thyroid tissue and benign lesions, but its expression is reduced with the progression of differentiated TC towards more aggressive, undifferentiated lesions (reviewed in [21]). In view of our findings, one could speculate that by compromising cell–cell adhesion, this loss of E-cadherin in advanced TCs could contribute to precluding NIS PM residency. Coupled with a decreased transcriptional expression of the symporter, namely via the hyperactivation of the MAP kinase pathway through NRAS and BRAF mutations [12,22], a reduced PM residency could be sufficient to promote RAI refractoriness in advanced TCs. Initial studies towards RAI resensitization therapy in refractory TC have highlighted that approaches promoting TC epithelial redifferentiation often led to improvements, albeit partial, in RAI uptake by TC cells in vitro and in vivo [23]. In particular, treatment with retinoic acids (RA), biologically active metabolites of vitamin A, were shown to improve the expression of E-cadherin and several other epithelial markers in TC cells, promoting cell adhesion [24]. In addition, pilot clinical studies have shown that iodide uptake can be re-stimulated after RA treatment in 20–50% of patients with RAI refractory TC [23,24,25]. Currently, RAI resensitization approaches have been focused on improving NIS transcriptional expression, which is downregulated in most TCs, through the inhibition of multiple components of the MAPK pathway, individually or in combination [26]. However, our findings provocatively suggest that a combination of MAPK inhibitors with agents that would improve cell–cell adhesion in refractory TC cells could potentially be of added clinical benefit.

## 5. Conclusions

In light of our findings, it will be important to clarify in future studies if the positive response of refractory TC to agents such as RA is associated with improved cell–cell adhesion and NIS PM residency and whether this can be combined with drugs that enhance NIS expression to more successfully overcome RAI refractoriness.

## Figures and Tables

**Table 1:** Top five best-represented KEGG pathways among the NIS plasma membrane interactors described in [10]. The analysis was performed using STRING algorithm (https://string-db.org/, accessed on 5 October 2021).

KEGG Pathway Analysis			
Pathway ID	Description	Hit Count	FDR
4810	Regulation of actin cytoskeleton	12	1.84 × 10^−6^
4520	Adherens junction	8	1.84 × 10^−6^
5130	Pathogenic Escherichia coli infection	7	3.22 × 10^−6^
4530	Tight junction	8	7.13 × 10^−5^
5131	Shigellosis	6	7.13 × 10^−5^

## Data Availability

The data are contained within the article or Supplementary Materials.

## Supplementary Materials

The following are available online at https://www.mdpi.com/article/10.3390/cancers14215362/s1. Figure S1: STRING-generated protein network of the candidate NIS interactors clustering in the top 2 KEGG pathway categories; Figure S2: Immunofluorescence labelling of HA-NIS-TPC1 cells treated for 30 min with either CaCl2 (1mM)-containing PBS (A) or calcium-free PBS containing 4 mM of EGTA (B). Grey dotted-line squares, within the full RGB images to the left, delimitate regions of interest 2x magnified in the image to the right and split below into the red (HA-NIS labeling with a rabbit anti-HA antibody, followed by Alexa542-conjugated anti-rabbit), green (actin labeling with FITC-conjugated phalloidin), and blue (mouse anti-E-cadherin antibody, followed by Alexa405-conjugated anti-mouse) channels. Solid white lines in the full RGB images represent 50 μm. White arrows indicate regions of co-localization either between NIS, E-cadherin and actin at cell-cell contacts (A) or between internalized E-cadherin and NIS at the cells’ cytoplasm (B). File S1: Original WB films.
