# Supplementary material for: Adherens Junction Integrity Is a Critical Determinant of Sodium Iodide Symporter Residency at the Plasma Membrane of Thyroid Cells
Source: Cancers (Basel). 2022 Oct 31;14(21):5362. doi: 10.3390/cancers14215362 (PMC9659096; doi:10.3390/cancers14215362)
Supplement: Supplementary file 1 [file cancers-14-05362-s001.zip › figures.pdf]

Adherens Junction integrity is a critical determinant of sodium iodide symporter residency at the plasma membrane of thyroid cells

Márcia Faria [1,2,3], José Vareda [1,4], Micaella Miranda[1,4] , Maria João Bugalho [1,5], Ana Luísa Silva [1,4,5,†,*], Paulo Matos [2,3,†,]

1 Serviço de Endocrinologia, Diabetes e Metabolismo do CHULN-Hospital Santa Maria, 1649-028 Lisboa, Portugal

2 BioISI-Biosystems and Integrative Sciences Institute, Faculdade de Ciências da Universidade de Lisboa, 1749-016 Lisboa, Portugal

3 Departamento de Genética Humana, Instituto Nacional de Saúde Doutor Ricardo Jorge, 1649-016 Lisboa, Portugal

4 ISAMB-Instituto de Saúde Ambiental, Faculdade de Medicina da Universidade de Lisboa, 1649-028 Lisboa, Portugal

5 Faculdade de Medicina da Universidade de Lisboa, 1649-028 Lisboa, Portugal

* Correspondence: silva.r.analuisa@gmail.com

† Co-senior authors.

Supplementary Figures

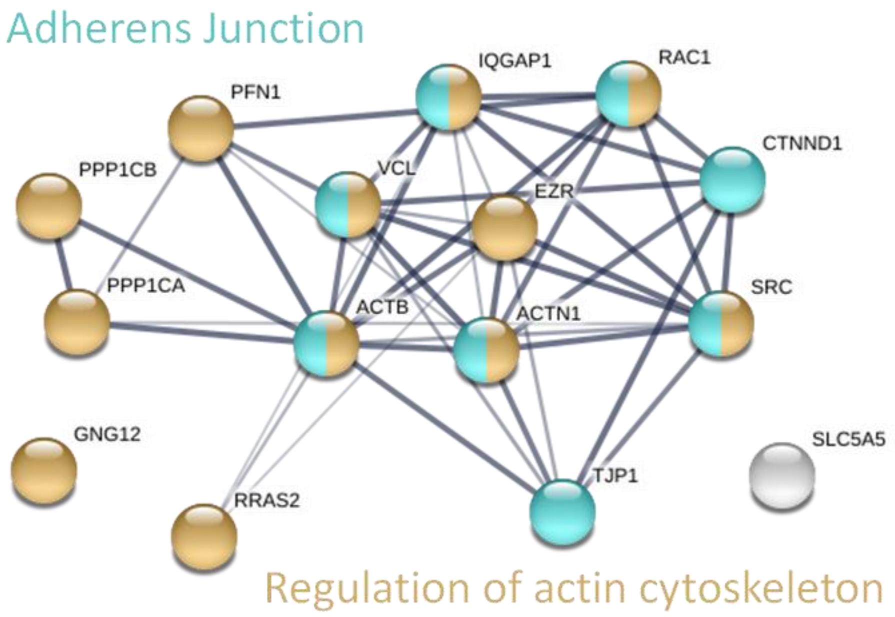

Supplemental Figure S1: STRING-generated network depicting predicted associations between the 14 high confidence NIS candidate interactors at the plasma membrane that cluster in the two top ranking KEGG pathway categories. Network nodes represent the identified proteins. The grey lines connecting two nodes represent protein associations extrapolated from textmining-, experimental- and database-collected evidence. The thickness of the lines is proportional to the degree of confidence for the predicted association between nodes. Cian and gold overlay colors indicate proteins clustering under “Adherens Junction” and “Regulation of actin cytoskeleton” KEGG pathway terms, respectively (see also Table 1).

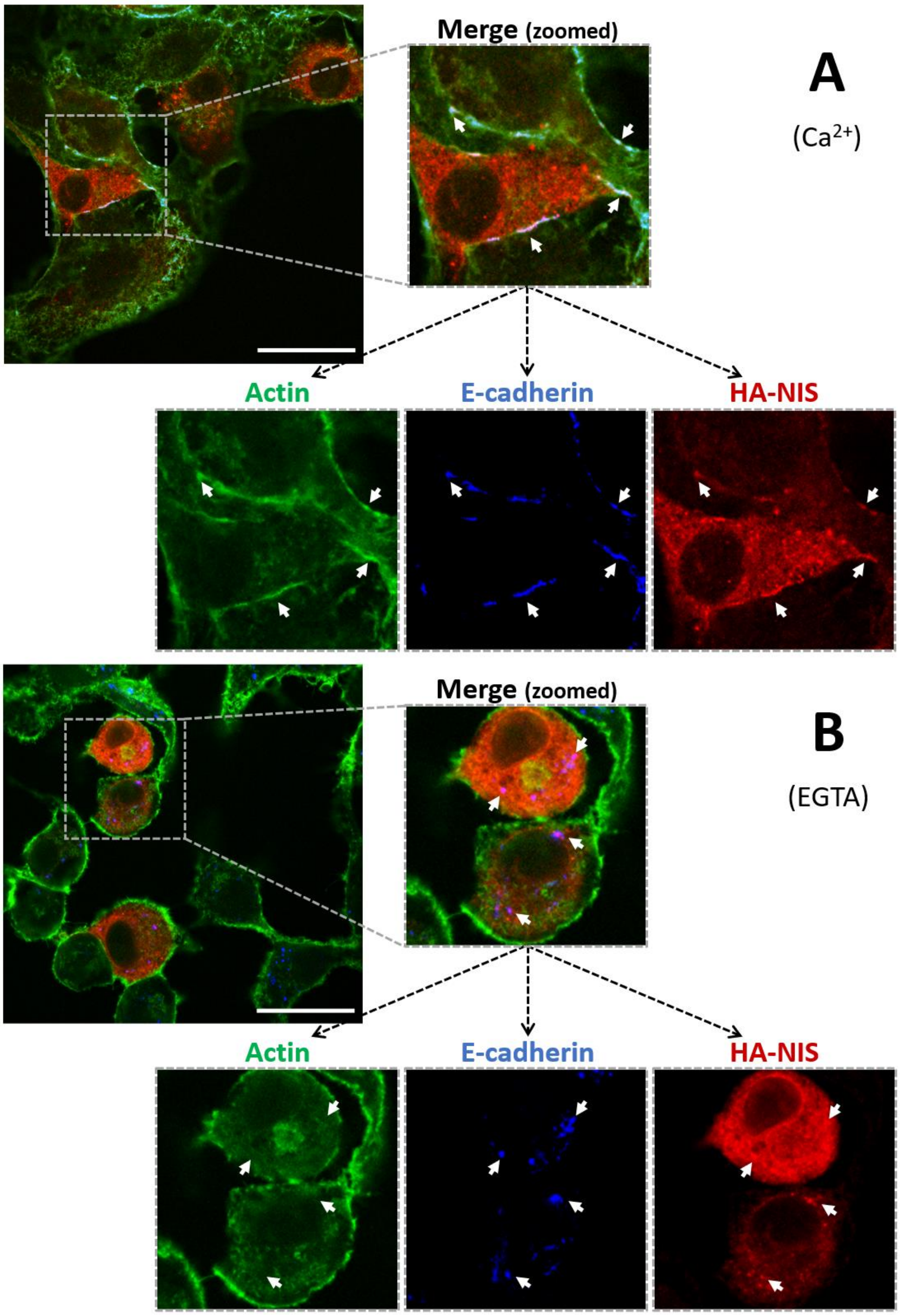

Supplemental Figure S2: Immunofluorescence labelling of HA-NIS-TPC1 cells treated for 30 min with either $CaCl_2$ (1mM)-containing PBS [A] or calcium-free PBS containing 4 mM of EGTA [B]. Grey dotted-line squares, within the full RGB images to the left, delimitate regions of interest 2x magnified in the image to the right and split below into the red (HA-NIS labeling with a rabbit anti-HA antibody, followed by Alexa542-conjugated anti-rabbit), green (actin labeling with FITC-conjugated phalloidin), and blue (mouse anti-E-cadherin antibody, followed by Alexa405-conjugated anti-mouse) channels. Solid white lines in the full RGB images represent 50 µm. White arrows indicate regions of co-localization either between NIS, E-cadherin and actin at cell-cell contacts [A] or between internalized E-cadherin and NIS at the cells' cytoplasm [B].
