# Supplementary material for: Adherens Junction Integrity Is a Critical Determinant of Sodium Iodide Symporter Residency at the Plasma Membrane of Thyroid Cells
Source: Cancers (Basel). 2022 Oct 31;14(21):5362. doi: 10.3390/cancers14215362 (PMC9659096; doi:10.3390/cancers14215362)

Adherens Junction integrity is a critical determinant of sodium iodide symporter residency at the plasma membrane of thyroid cells

Márcia Faria [1,2,3], José Vareda [1,4], Micaella Miranda[1,4], Maria João Bugalho [1,5], Ana Luísa Silva [1,4,5,†,*], Paulo Matos [2,3,†,]

,

1 Serviço de Endocrinologia, Diabetes e Metabolismo do CHULN-Hospital Santa Maria,

1649-028 Lisboa, Portugal

2 BioISI-Biosystems and Integrative Sciences Institute, Faculdade de Ciências da Universidade de Lisboa, 1749-016 Lisboa, Portugal

3 Departamento de Genética Humana, Instituto Nacional de Saúde Doutor Ricardo Jorge,

1649-016 Lisboa, Portugal

4 ISAMB-Instituto de Saúde Ambiental, Faculdade de Medicina da Universidade de Lisboa,

1649-028 Lisboa, Portugal

5 Faculdade de Medicina da Universidade de Lisboa, 1649-028 Lisboa, Portugal

* Correspondence: silva.r.analuisa@gmail.com

† Co-senior authors.

Original WB films used to assemble the various Figures

Figure 1C

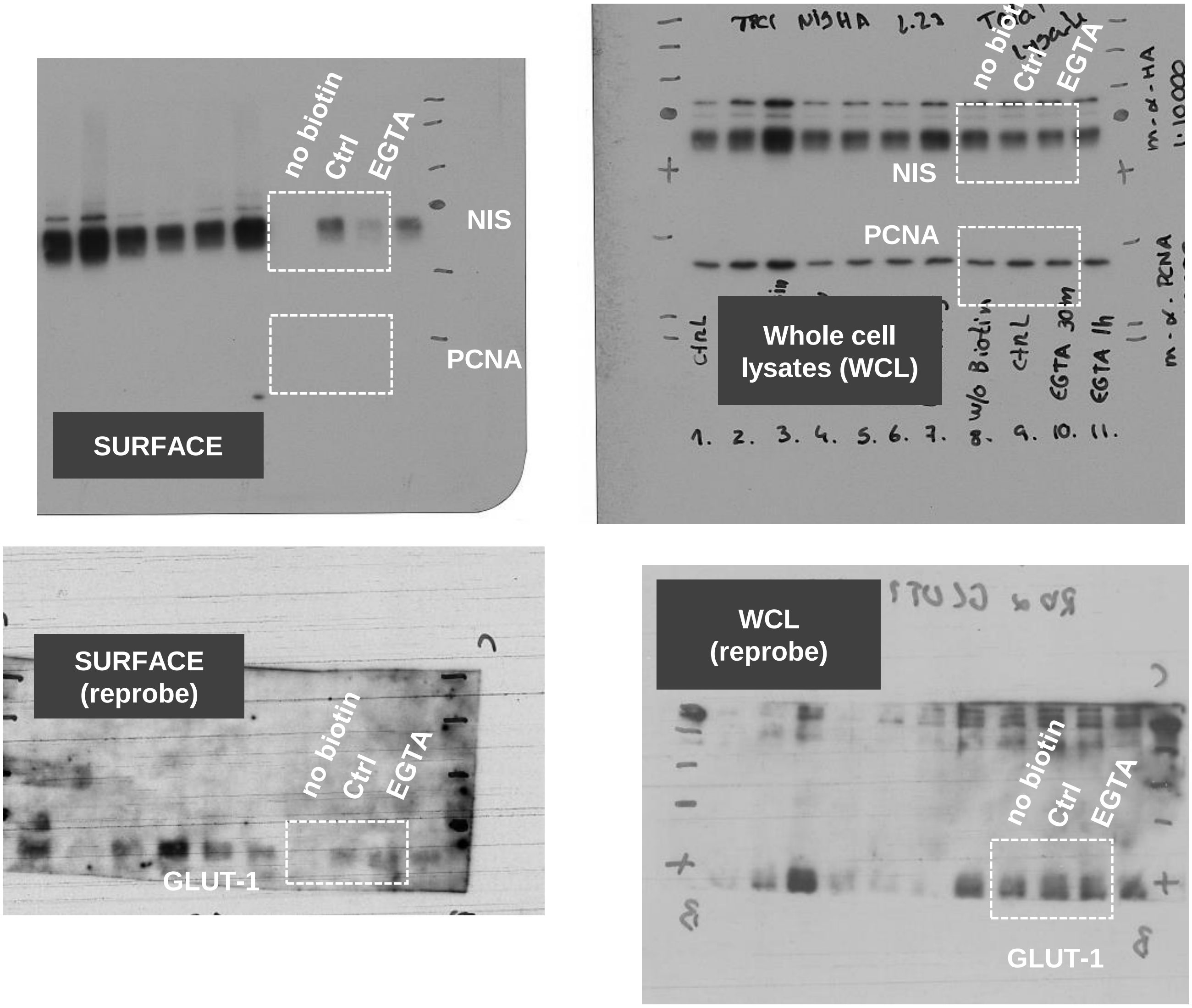
no biotin
Ctrl
EGTA
NIS
PCNA
SURFACE
NIS
PCNA
Whole cell lysates (WCL)
SURFACE (reprobe)
GLUT-1
WCL (reprobe)
GLUT-1

Figure 2A

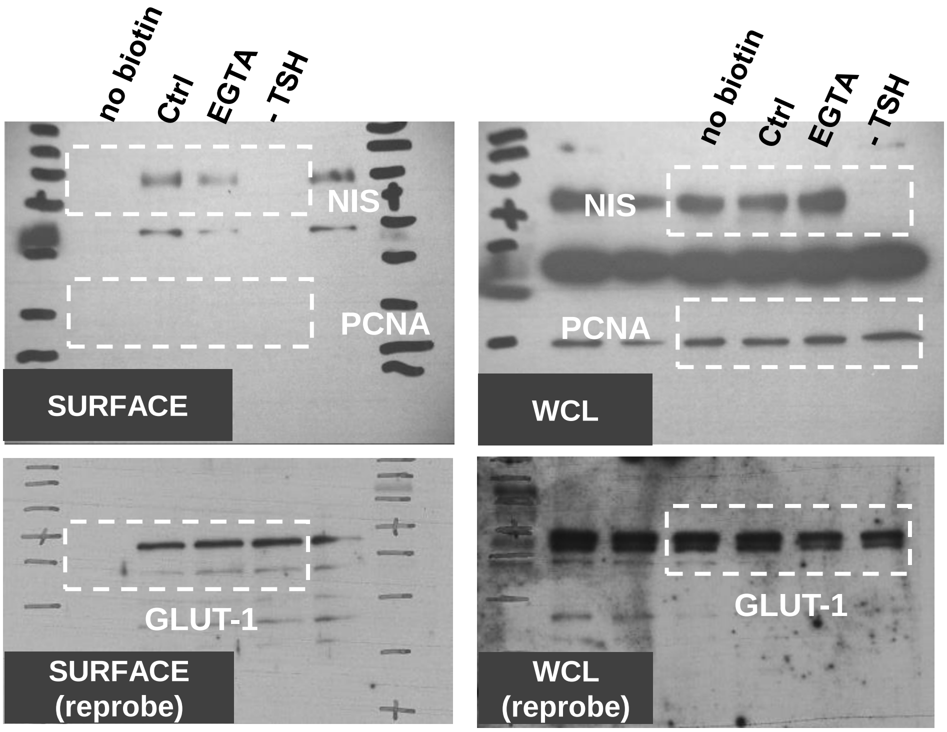

no biotin
Ctrl
EGTA
- TSH
NIS
PCNA
SURFACE
no biotin
Ctrl
EGTA
- TSH
NIS
PCNA
WCL
GLUT-1
SURFACE (reprobe)
GLUT-1
WCL (reprobe)

Figure 3C

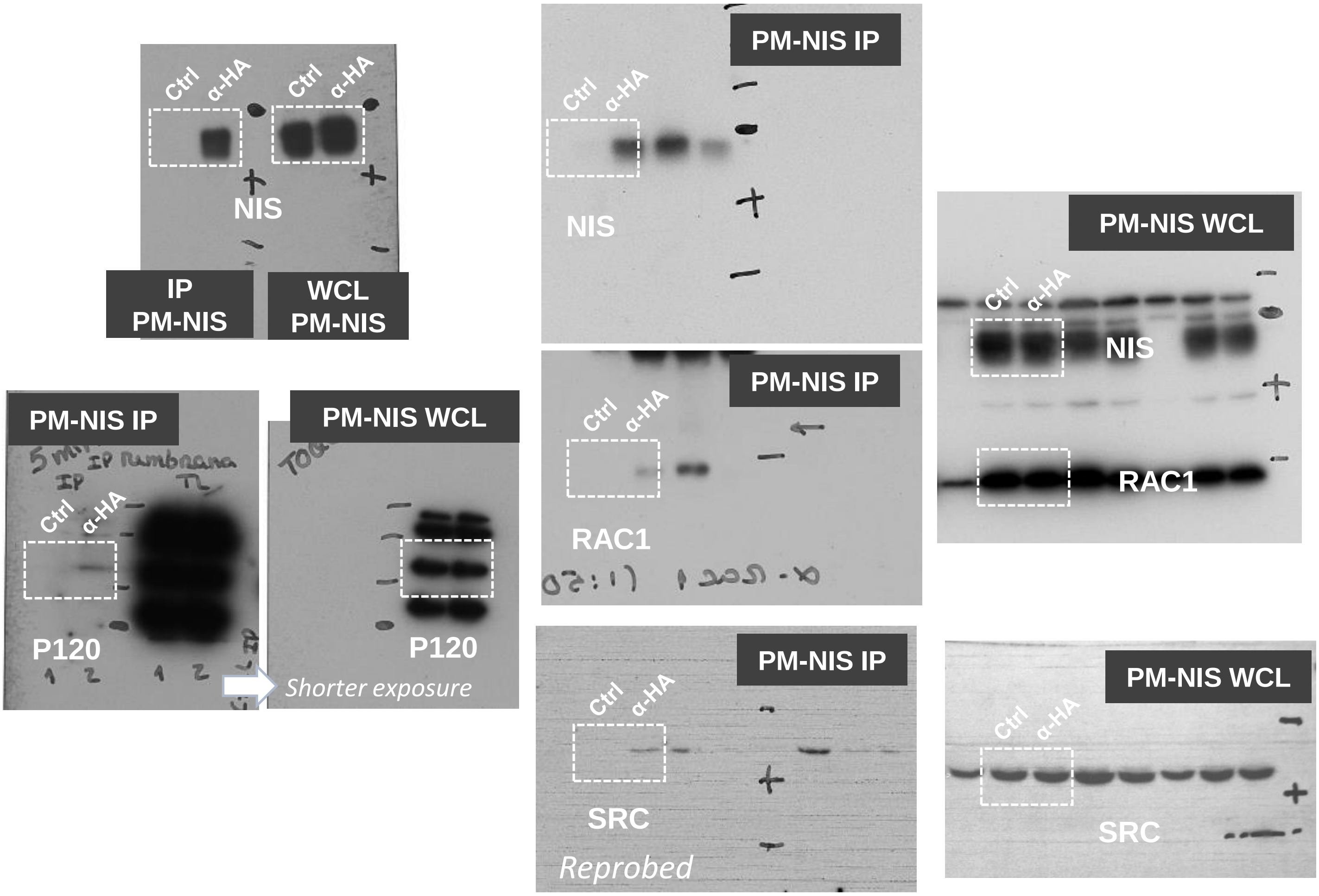

Figure 3D

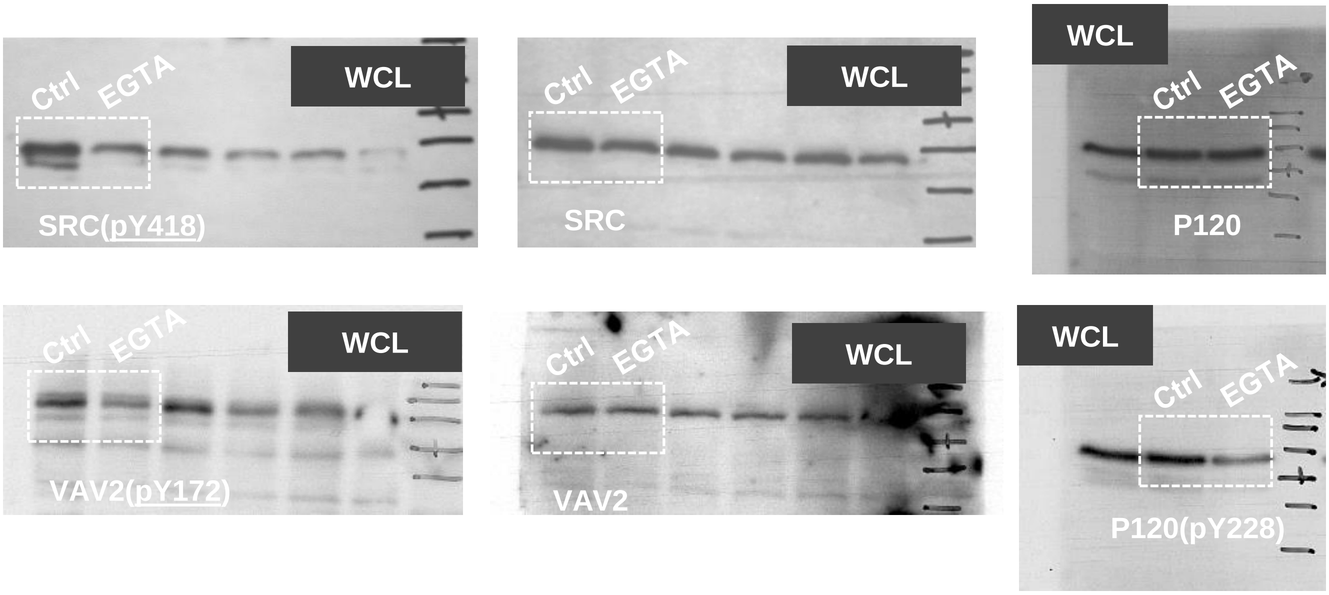

Figure 4A

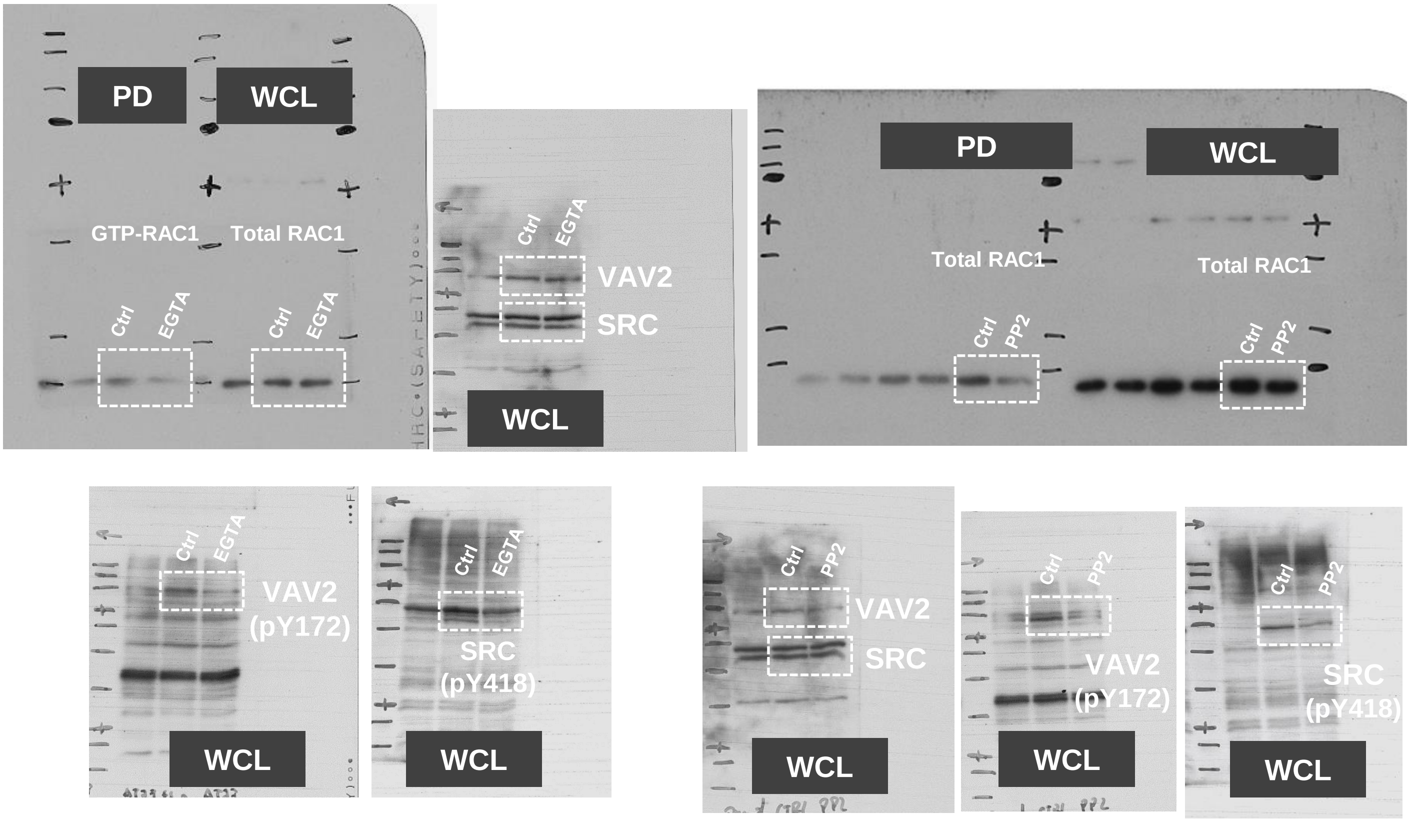

Figure 4C

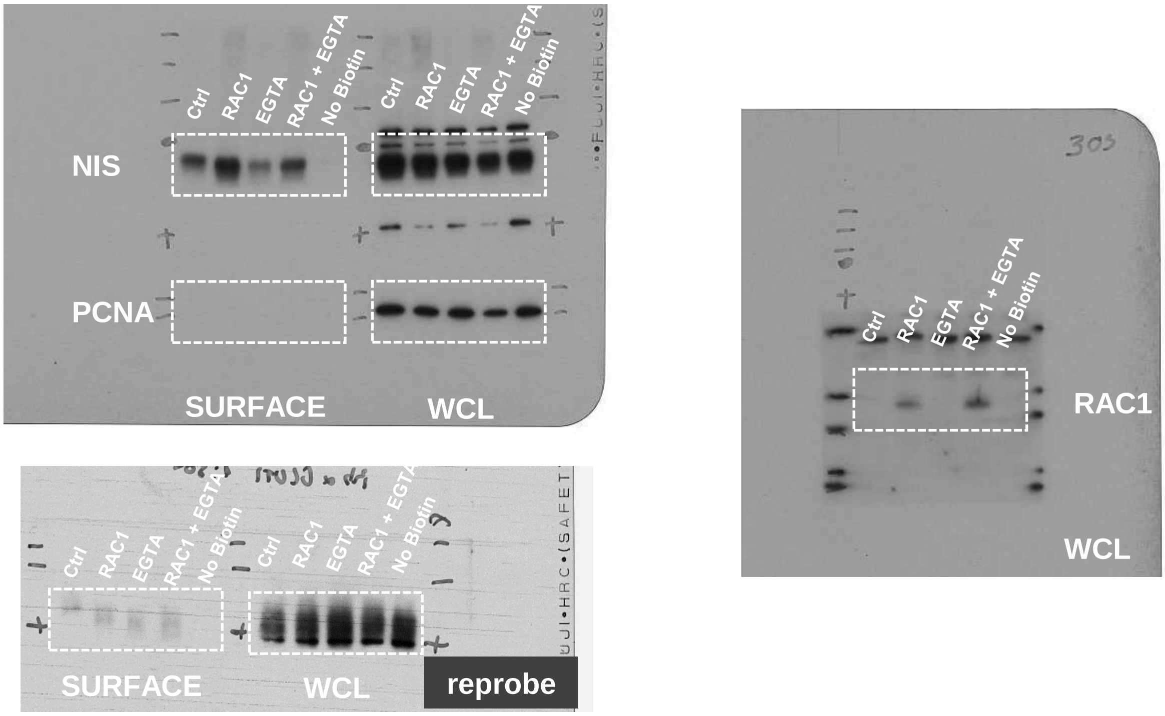

Figure 5A

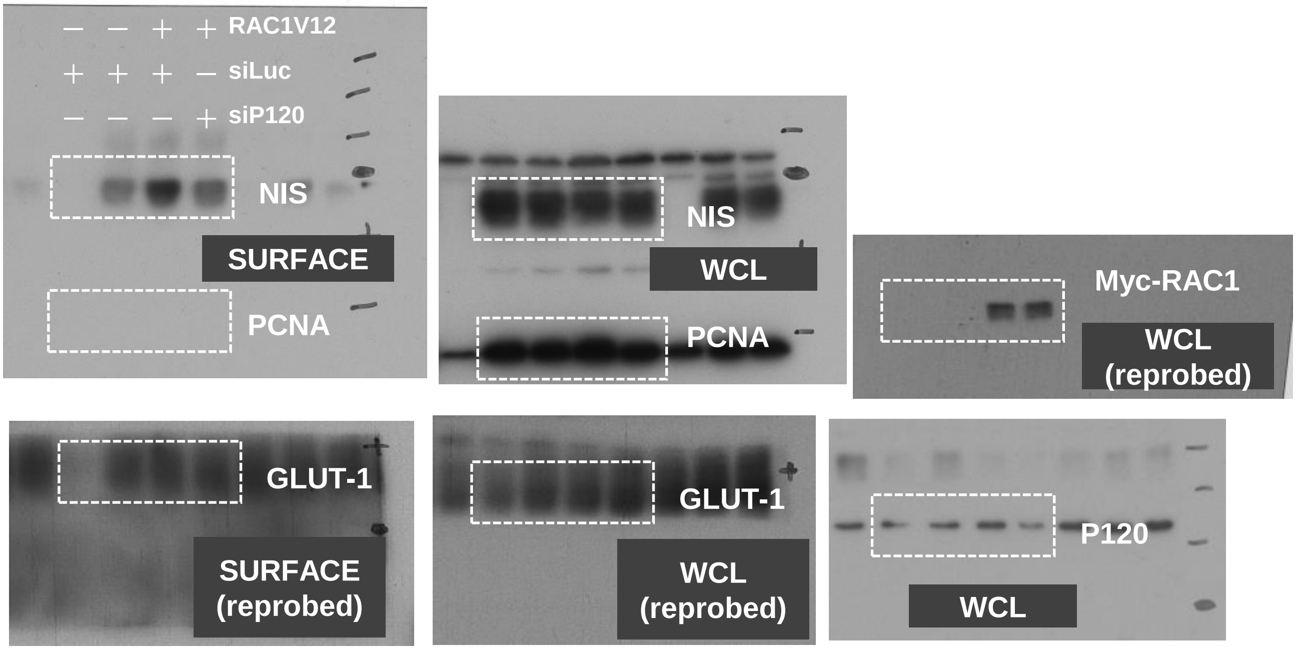

Figure 5B

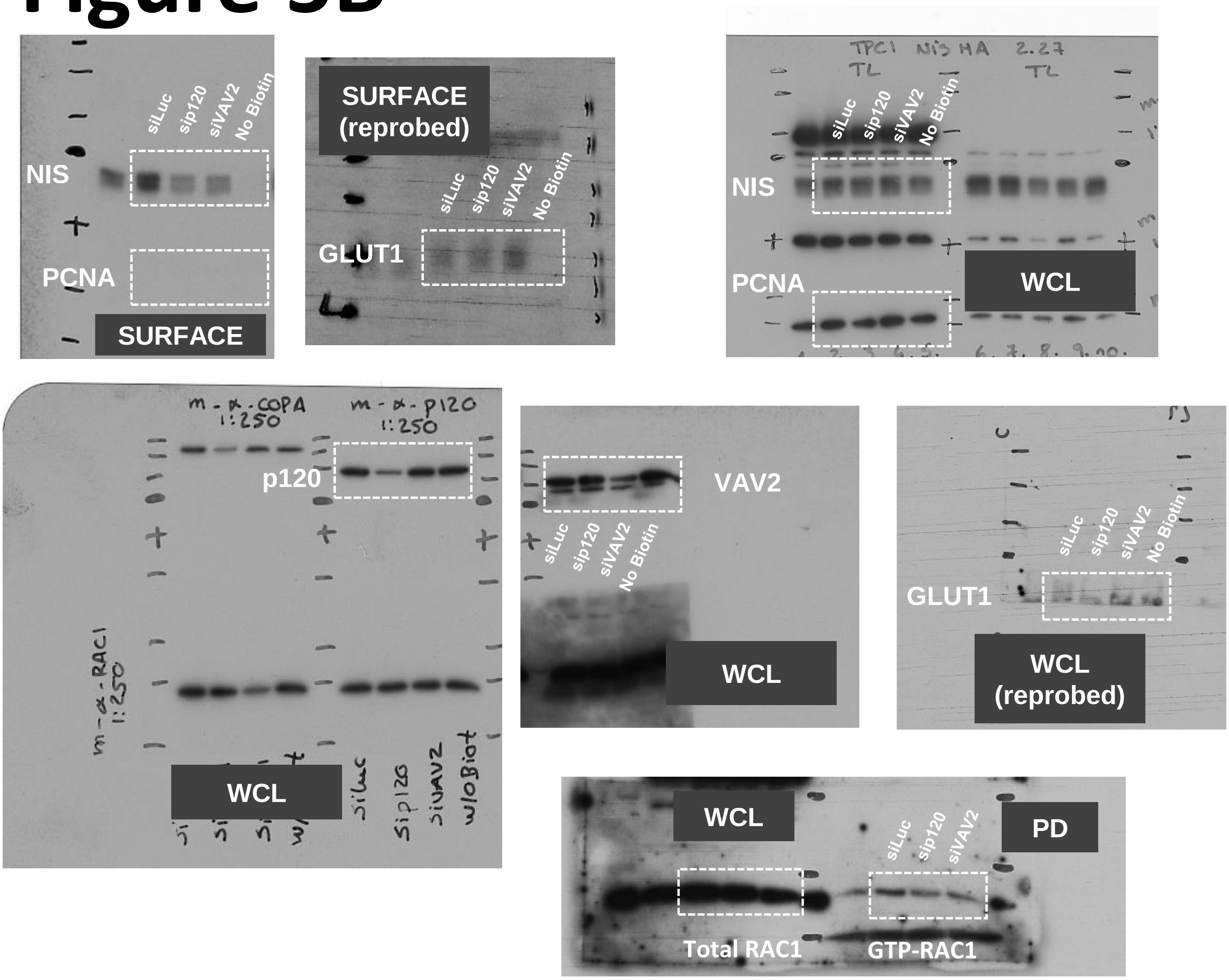

Supplement: Supplementary file 1 [file cancers-14-05362-s001.zip › File S1. Original WB films.pdf]
